# Items

**Published:** 1881-11

**Authors:** 


### Items.

---

THE GENERAL ASSEMBLY has appropriated $165,000 for the enlargement and improvement of the State Asylum for the insane at Midway.

This extraordinary appropriation was made necessary by the crowded condition of the present building, and the demand for additional accommodations, many applicants having been refused admission for lack of room.

A portion of this appropriation will also be used to provide better and more generous means for the "moral treatment" of the patients.

---

SOME OF OUR ESTEEMED EXCHANGES are fretting because their choice articles are appropriated by other journals without due credit. They apparently fail to appreciate evidences of appreciation. Let them try the effect of publishing editorials of less merit, and contributions of diminished excellence.

---

THE *London Lancet* and the Atlanta MEDICAL REGISTER will be sent to any address one year on receipt of $5.00. See prospectus of the Lancet elsewhere.

---

MR. HOLMES, it is said, is very indignant about the publication of the American edition of his system of surgery. It seems that there is no international copy right law, and the arrangements for issuing the work were all perfected, so he claims, without his knowledge or consent.

---

LET EVERY MEDICAL PRACTITIONER within this State remember that he is required by law to register in the office of the clerk of the superior court of the county wherein he resides, on or before the first day of December, 1881, his name, residence, place of birth and under what authority he is practicing, and when and by whom such authority was granted.

---

THE NORTHWESTEN LANCET is the name of a new bi-weekly medical journal, published at Saint Paul, Minnesota, and edited by Jay Owens, M.D.

A vacancy in the field of medical journalism was discovered in the "new northwest," and the *Lancet* proposes to fill it.

---

THE medical colleges of this city were opened for the winter session about the middle of October, with a fair number of students in attendance. The session of the Atlanta Medical College was inaugurated by an introductory lecture from Prof. J. S. Todd; the session of the Southern Medical College with an address by Prof. Jno. Thad. Johnson.

---

Dr. T. Gaillard Thomas has resigned the Chair of Obstetrics and Gynecology in the College of Physicians and Surgeons, New York. It is stated that he will deliver a special series of lectures on uterine displacements. Dr. James W. McLane was appointed to the vacated chair, and Dr. Paul F. Munde was appointed clinical lecturer on the diseases of women.

---

At the sixth annual meeting of the American Gynecological Society, just held in New York, Dr. T. Addis Emmet, of New York, was elected President, and Dr. G. H. Lyman, of Boston, and Dr. E. Noeggerath, of New York, were elected Vice Presidents. Dr. Henry F. Campbell, of Augusta, read a paper on "Erysipelas in Childbed Without Pueperal Peritonitis." A number of interesting papers were presented. The next annual meeting will be held in Boston, September, 1882.

---

We have examined the announcement of "Guy's Hospital School," London. It is a pamphlet of 80 pages, and presents a more formidable appearance than the most pretentious announcements of our American colleges.

---

Atlanta may now properly be called a medical centre. It boasts of four medical colleges; two of these are regular, one eclectic, and one "reformed eclectic." We give the name, but not the definition of the last.

---

The *Medical Annals* presents a case of "Maggots in the Middle Ear." On looking into the details, we can only find that a number of the larvæ were extracted (by forceps), not from the middle ear, but from the external auditory canal—a not very uncommon occurrence.

---

Dr. C. W. Nutting, formerly of this city, who removed to Etna, California, several years since, was married in that State October 3d. He has many warm friends who will rejoice to know that he has prospered in his new home.

---

Dr. James P. White, of Buffalo, N. Y., is dead.

